# Novel miniaturized fluorescence loop-mediated isothermal amplification detection system for rapid on-site virus detection

**DOI:** 10.3389/fbioe.2022.964244

**Published:** 2022-08-24

**Authors:** Yanqi Wu, Liping Bai, Chengfu Ye, Yuhong Guan, Kunming Yan, Hui Chen, Zhihong Jiang

**Affiliations:** ^1^ State Key Laboratory of Quality Research in Chinese Medicine, Macau University of Science and Technology, Taipa, China; ^2^ Shenzhen LemnisCare Medical Technology Co., Ltd, Shenzhen, China; ^3^ Hunan Key Laboratory of Biomedical Nanomaterials and Devices, Hunan University of Technology, Zhuzhou, China

**Keywords:** LAMP, POCT, norovirus, fluorescence detection, aerosol pollution

## Abstract

New pathogen outbreaks have progressed rapidly and are highly infectious in recent years, increasing the urgency of rapid and accurate detection of pathogenic microorganisms. Based on the point-of-care testing (POCT) requirements, in this study, a real-time fluorescent loop-mediated isothermal amplification (LAMP) detection system was developed and applied to pathogen detection. The system is compact and portable, with good uniformity and reproducibility, and it can detect pathogens rapidly and effectively. For norovirus detection, the linear range was 10^0^–10^6^ copies/μL. The system can achieve the theoretical sensitivity of LAMP detection, conclusions could be obtained within 35 min, and quantitative detection was possible. The test results of 45 clinical samples were consistent with quantitative PCR (qPCR) and clinical results, and the accuracy could reach 100%. This system has the characteristics of portability, speed, and POCT accuracy, and the cost is much lower than that of commercial qPCR. Therefore, it is suitable for remote areas or places with relatively poor conditions and environments requiring on-site conditions. It can also be widely used to detect various epidemics and unexpected diseases.

## 1 Introduction

In recent years, due to the continuous emergence of new pathogens such as Zika virus, Ebola virus, SARS, and COVID-19 etc. (Liao et al., 2017; Al-Saud et al., 2020), rapid and highly infectious outbreaks of new pathogens have made rapid and accurate identification of pathogenic microorganisms and diagnosis of infectious disease an urgent need (Chen et al., 2018; Demrba et al., 2021). The technology of diagnosing diseases has been developing continuously, from the earliest culture method to hematological analysis, specific protein detection, immunological antigen, and antibody detection, to pathogen mass spectrometry and nucleic acid analysis (Hussain et al., 2020) six; the methods are becoming increasingly accurate. Compared with traditional etiological diagnostic methods with higher detection values but a slower detection process, nucleic acid testing is currently the leading technology for pathogen diagnosis (Tang et al., 2020; Fang et al., 2021; Xu et al., 2021).

The application of the mainstream polymerase chain reaction (PCR) and its derivative technologies in various nucleic acid testing is unsuitable for on-site application due to high instrument requirements and complicated operations (He et al., 2017). Various high-efficiency, high-sensitivity, and specific isothermal amplification technologies can meet the needs of on-site detection, such as low equipment requirements and simple operation (Chen et al., 2017; Liu et al., 2019). Presently, the main isothermal amplification technologies are rolling circle amplification (RCA) (Gu et al., 2018; Zhao et al., 2020), loop-mediated isothermal amplification (LAMP) (Chen et al., 2018), strand displacement amplification (SDA), nucleic acid sequence-based amplification (NASBA), helix-dependent amplification (HAD), and recombinase polymerase amplification technology (RPA) (Knox and Beddoe, 2021; Wei et al., 2022). Although these technologies have their limitations, LAMP is currently the most widely used. LAMP technology enables simple and rapid nucleic acid amplification. Due to its extremely high amplification efficiency, several products and byproducts are generated in a short time, which is more convenient for detection. Common detection methods include gel electrophoresis, turbidity, colloidal gold test strip, metal ion indicator, and fluorescence detection methods. The principle of fluorescence detection is the same as that of real-time fluorescent PCR. Through fluorescent probes or dyes, the amplification speed of LAMP is more than that of PCR and LOD of LAMP method is theoretically comparable to the real-time fluorescent PCR method.

On the other hand, with the advancement of medical means and the continuous improvement of the degree of automation, society has paid more attention to the usage scenarios of point-of-care testing (POCT) in recent years (Chen et al., 2019; Liu et al., 2022). The core of this concept is that the tests that can only be performed in laboratories or hospitals can be transferred to people’s homes, being a part of their daily lives, without the need for professional personnel to operate, simplifying testing procedures, reducing testing cost and time, and at the same time, meeting the requirements of accuracy. However, this concept requires a high degree of integration and automation of medical instruments, as well as good portability. Compared with the cumbersome process of the traditional clinical laboratory and the inability to implement rapid on-site testing, POCT realizes the miniaturization of the instrument, the simplicity of operation, and the real-time testing on-site. The fluorescent LAMP detection method combined with the POCT detection instrument can realize the rapid, accurate, and efficient detection of pathogen nucleic acid in remote areas or places with relatively poor conditions. Many businesses are dedicated to the research of POCT systems, high accuracy, miniaturization, and portability are their consistent goals. For example, ABBOTT specializes in point-of-care solutions and its ID NOW™ isothermal nucleic acid amplification detector can simultaneously detect influenza A and B viruses within 15 min without the need for expensive laboratory equipment (Masciotra et al., 2013; van den Berk et al., 2003). Lucira’s Lucira COVID-19 all-in-one test kit utilizes RT-LAMP technology to detect the N gene RNA of SARS-CoV-2, generating a signal from several RNA copies in less than 30 min, generated by the process of amplification A change in pH, followed by a change in the color of the dye in the reaction mixture, enables accurate self-diagnosis at home (2021). The Cue™ COVID-19 Test Cartridge Pouch developed by Cue Health can produce test results in 20 min and transmit the results to the smart devices of users and doctors (2021). Research shows that the product has a sensitivity and specificity of up to 98%. Among these products, methods such as immunochromatography, LAMP and Real-time PCR are used to achieve rapid detection of individual samples, and the reagents and consumables can be discarded for one-time use. The above-mentioned platforms are very fast and convenient for POCT applications, but for some remote or under-condition areas or places, the cost of a single detection of these instruments increases due to the specificity of consumables or reagents; on the other hand, the small quantitative testing is often also required for POCT. Therefore, we hope to design a rapid detection system with low throughput and strong versatility.

In order to meet the needs of on-site nucleic acid detection of a small number of samples, we developed a portable fluorescent LAMP detection system that can rapidly and effectively detect pathogens. Herein, after introducing the system structure and evaluating and verifying its performance, we applied the system to actual sample detection. Compared with those of laboratory detection methods, the results show that the system’s accuracy, sensitivity, and stability can meet the needs of nucleic acid testing and enable early diagnosis of pathogens on the spot, providing an effective basis for disease prevention, control, and precise treatment.

## 2 System structure

**FIGURE 1:**
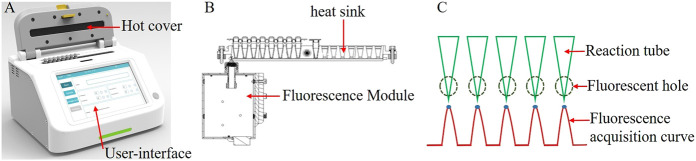
**(A)** Overall schematic of the system; **(B)**. Schematic of the heat trap and fluorescence modules; **(C)**. Multi-channel fluorescence signal acquisition curve.

Overall system dimensions: The portable fluorescent LAMP detection system has a length, width, and height of 240 × 180 × 150 mm, respectively, and a weight of 3.6 kg, with an internally integrated heat sink, heating module, fluorescent module, and display module (Figure 1A). There are two eight-row heat sink holes, which can detect 16 samples simultaneously. Unlike the commercial Roche and ABI fluorescence detection instruments, which read the fluorescence from the top, this system reads the fluorescence from the side. By opening a 3-mm small hole on the lower side of each heat sink hole, the fluorescence module collects and detects the fluorescence signal when it passes through each well by sliding, as shown in Figure 1B. At present, we have tested the fluorescent channel suitable for SYBR/FAM, which can be detected by their similar fluorescent probes or dyes. The collected fluorescence signal will have a maximum fluorescence value in each well, and this maximum value is selected as the fluorescence value of the well in this reaction time period, as shown in Figure 1C. The heating module is mainly made up of a heat sink and heat cover heating. The heat sink heating adopts a thermoelectric cooler, while the heat cover heating adopts a thin film heater. The display module integrates a human–computer interaction interface through which the user can set the experimental parameters, including reaction temperature, time, sample well information, etc., and display the fluorescence detection curve in real-time during the experiment.

## 3 System performance

The main factors that affect the experimental results are temperature, fluorescence signal acquisition, etc. Therefore, it is necessary to evaluate the performance of the entire system from hardware and experiments.

### 3.1 Temperature

**FIGURE 2:**
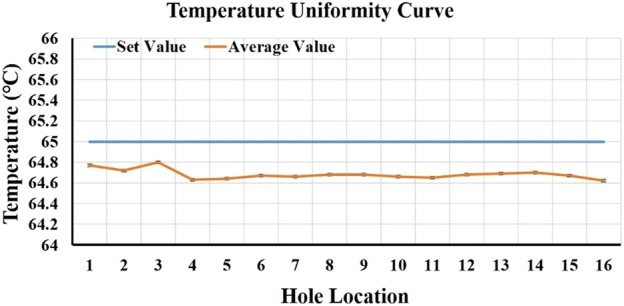
16-well temperature uniformity curve.

Temperature, especially its uniformity, stability of the heat sink, and the heating speed, are important factors that affect the amplification effect of LAMP. To test the temperature of 16 sample wells, two eight-row 0.2 ml centrifuge tubes (Aixin Biotechnology (Hangzhou) Co., Ltd, Hangzhou, China) were placed in a heat trap, 50 μL of pure water was added to each centrifuge tube, and the heat sink temperature was set to 65°C. The temperature of the liquid in each centrifuge tube was then measured. Each hole was tested every 5 s for 10 consecutive times. We analyzed the average temperature, standard deviation (SD), coefficient of variation (CV) values, etc., of each hole. The test results are depicted in Figure 2. It shows that among the 16 holes, the minimum average temperature was 64.62°C, and the maximum was 64.8°C, the temperature fluctuation of 10 measurements per well was less than 0.3°C, the maximum temperature difference from the set temperature did not exceed 0.5°C, and the CV value was 0.07–0.15%. The setting temperature was 27–65°C, and it took 15 s to reach the set temperature for the first time, indicating that the heating rate can reach 2.53°C/s. The temperature test results revealed that the temperature uniformity of the 16 wells was good, the temperature control was stable, and the heating rate was fast, which did not affect the amplification.

### 3.2 Evaluation and verification of RT-LAMP performance

To test the usability of the system, the sensitivity, detection limit, uniformity, and repeatability stability of the system were tested and evaluated and compared with commercial qPCR instruments Gentier 96E (Xi’an Tianlong Technology Co., Ltd, Xi’an, China), and the results were compared with qPCR detection. The LAMP and qPCR reagents were purchased from commercial kits (Shenzhen LemnisCare Medical Technology Co., LTD, Shenzhen, China). Table 1 describes the reagents and conditions for RT-LAMP and RT-qPCR detection.

**TABLE 1:** RT-LAMP and RT-qPCR reaction conditions.

RT-LAMP		RT-qPCR	
Experimental components	Condition setting	Experimental components	Condition setting
10X Isothermal amplification buffer II, 2.5 μl		2x Buffer, 12.5 μl	50°C, 10 min 95°C, 5 min 95°C, 15 s 60°C, 30 s 45 cycles 60°C fluorescence collection
MgSO4 (100 mm), 1.5 μl		Enzyme mix, 1 μl	
dNTP Mix (10 mm), 3.5 μl	65 C 45 min,Fluorescence is collected every minute	Forward primer (20 μm), 1 μl	
FIP/BIP primers (40 μm), 1 μl		Reverse primer (20 μm), 1 μl	
F3/B3 primers (20 μm), 1 μl		Probe (10 μm), 1 μl	
LF/LB primers (20 μm), 1 μl		Nuclease-free water, 3.5 μl	
Nuclease-free water 7 μl		DNA or RNA Sample 5 μl	
NEB LAMP dye (50x), 0.5 μl			
Bst 3.0 DNA polymerase (8 U/μl), 2 μl			
DNA or RNA sample, 5 μl			
Total, 25 μl		Total, 25 μl	

### 3.2.1 Uniformity

We diluted the norovirus GⅡ standard to 10^4^ copies/*μ*L for the LAMP experiment, prepared 16 reaction solutions according to the experimental components in Table 1, distributed them into two eight-row 0.2 ml centrifuge tubes, put the centrifuge tubes into the system, set up the system according to the reaction conditions in Table 1, and observed the real-time fluorescence amplification curve of 16 wells. Simultaneously, we ran the same test on Gentier 96E (Xi’an Tianlong Technology Co., Ltd, Xi’an, China). The uniformity of the system was assessed by the time to threshold (Tt) values of the amplification curves and the coincidence of the curves.

### 3.2.2 Sensitivity and detection limit

We diluted the norovirus standard with sterile water in a 10-fold gradient to six concentrations (10^5^–10^0^copies/μL), configured the reaction solution according to Table 1, set a negative control, configured two copies of each concentration, and put them into the test system. Then, we configured the reaction solution according to the qPCR system in Table 1, added the same standard substance, and performed qPCR detection using the Tianlong real-time fluorescence PCR instrument. The detection limit of the system and the difference from qPCR detection were determined by amplification curve and Tt and Ct values.

### 3.2.3 Stability

Three different concentrations of standards were selected for testing, wherein the high, medium, and low concentrations were 10^5^copies/μL, 10^3^copies/μL, and 10^1^copies/μL, respectively. The reaction solution was configured according to the system in Table 1, a negative control was established, and three copies of each concentration were configured and introduced into the system for detection. The experiment was repeated thrice to detect the high, medium, and low concentrations. First, the stability was initially assessed by the amplification curve of the three experiments, and then the detection results of the three experiments were put together to compare the CV value of the average Tt. The Tt value refers to the calculation of the real-time fluorescent PCR Ct value, and the baseline was calculated according to the negative control fluorescence value of each experiment group.

#### 3.2.4 RT-LAMP and RT-qPCR for the detection of clinical samples

We collected 45 cases of norovirus diarrhea samples from the Guangdong Provincial People’s Hospital, performed nucleic acid extraction for clinical samples, adopted the magnetic bead method using a nucleic acid extraction kit (Shenzhen LemnisCare Medical Technology Co., LTD, Shenzhen, China), and performed the RT-LAMP test on this system. We set the reaction system and reaction conditions as stated in Table 1. Simultaneously, we conducted RT-qPCR test to compare the test results.

## 4 Results and discussion

### 4.1 Homogeneity assessment

**FIGURE 3:**
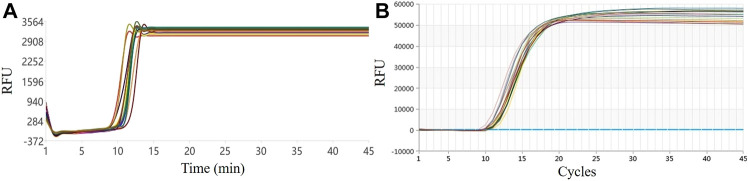
Six-well uniformity test results: **(A)** Our system. **(B)** Commercial qPCR instrument.

The 16-well LAMP real-time fluorescence amplification curve of this system and the amplification curve on a commercial qPCR instrument are shown in Figure 3. From the shape of the curve, the overlap of the two amplification curves is high. According to the Tt value, the average value of the reaction of 16 wells in this system was 12.56, the SD was 0.73, and the CV was 5.81%; the LAMP experimental results on a commercial qPCR instrument revealed an average Ct value of 9.83, an SD of 0.38, and a CV of 3.88%, as shown in Figure 3B. Compared with the commercial qPCR instrument, the CV was only 1.93% different, indicating that the overall uniformity of the system is good.

### 4.2 Sensitivity and detection linear range evaluation

**FIGURE 4:**
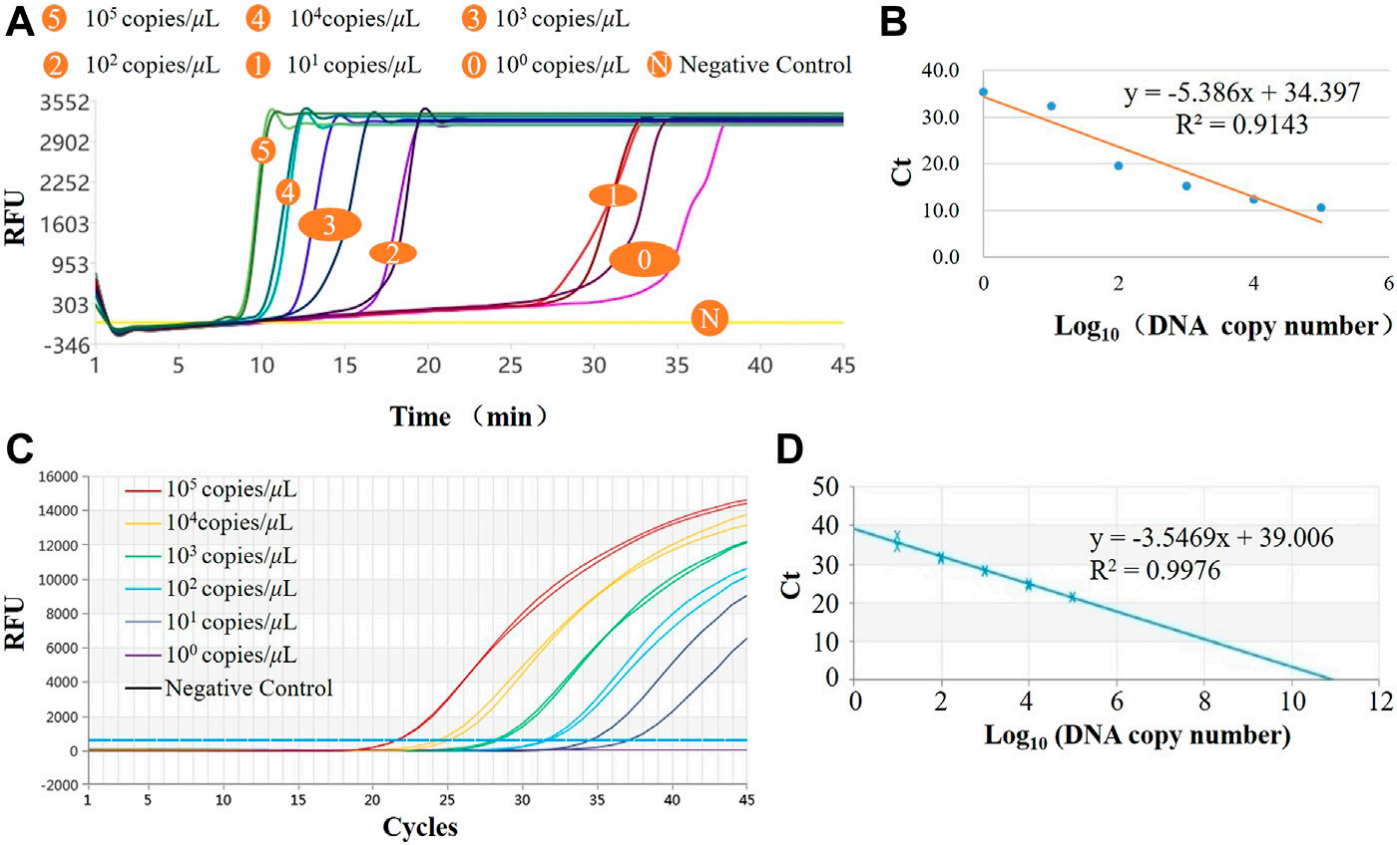
Results of norovirus sensitivity. **(A)** LAMP amplification curves of six concentration gradients in the system; **(B)** Standard curve for LAMP assay; **(C)** qPCR amplification curves of six concentration gradients in the system; **(D)** Standard curve for qPCR assay.

The LAMP amplification curves of six concentration gradients are shown in Figure 4A. From the shape of the amplification curve, it could be observed that the amplification efficiency was stable and high, and the sensitivity could reach 10^0^ copies/μL, i.e., copy numbers within 10 can be detected. The minimum detection limit detection time was <35 min. The logarithm of the plasmid concentration has an obvious linear relationship with the Tt value. As shown in Figure 4B, the *R* ^2^ value is 0.9143, indicating that the system also has potential quantitative performance. The real-time PCR amplification curves of the same six concentration gradients are shown in Figure 4C, and the standard curve is shown in Figure 4D. It could be observed from the amplification curve that the detection line of real-time fluorescent PCR was 10^1^ copies/μL, and the whole PCR process took about 90 min. From the perspective of the linearity of the standard curve, LAMP is worse than qPCR, which is primarily reflected in low concentration detection. Also, its amplification efficiency was not as good following high concentration detection, and the amplification was unstable, resulting in a decrease in the overall linearity, although it was basically a linear relationship. Quantitative detection was possible. In terms of detection time, LAMP was superior to the gold standard qPCR; In terms of sensitivity, theoretically qPCR assays can detect to <10 copies, which means that LOD (Limits of Detection) of LAMP and qPCR are comparable. This system uses the LAMP method for pathogen detection, which meets the actual needs in terms of accuracy and speed and is competitive in POCT.

### 4.3 Stability assessment

**FIGURE 5:**
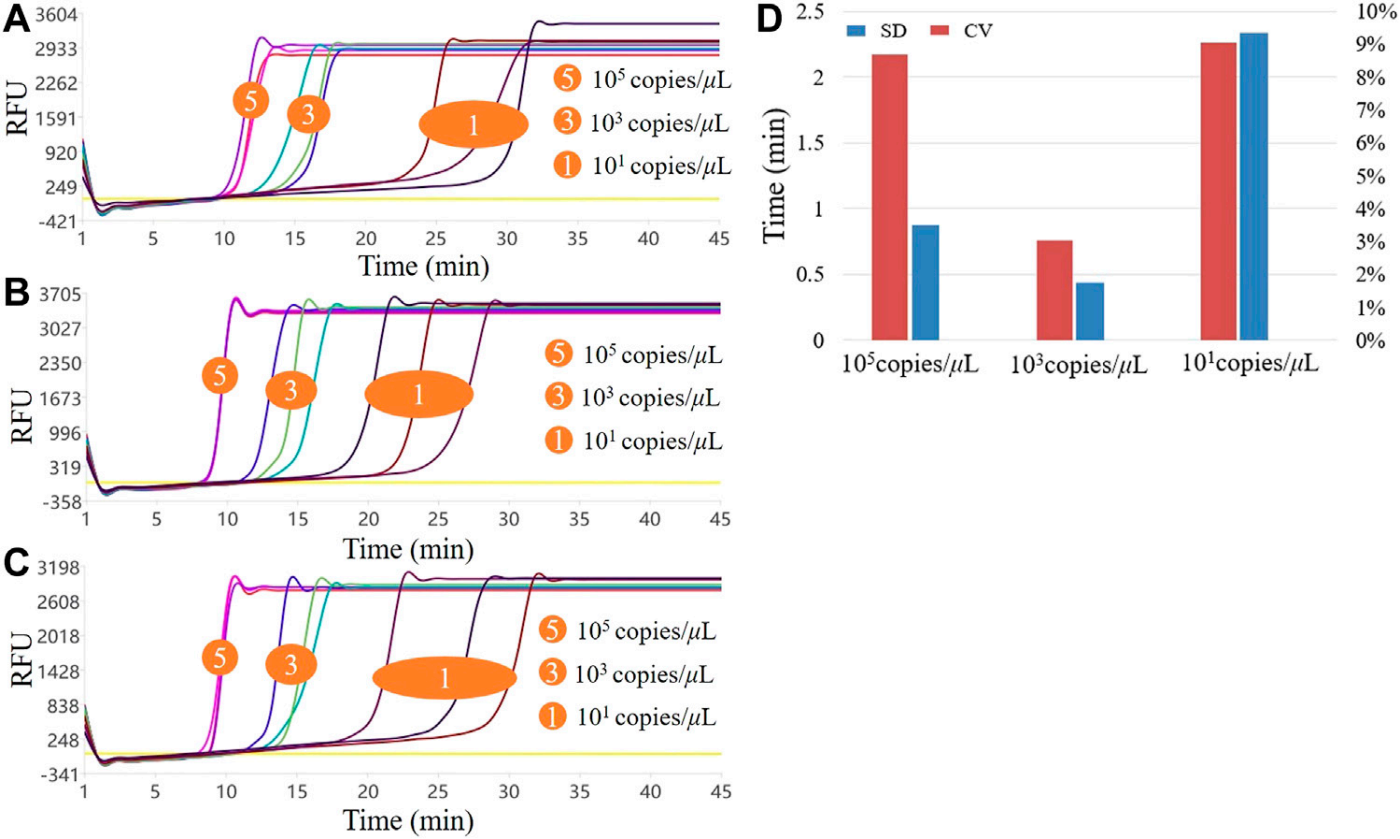
Results of the three LAMP experiments with high, medium, and low concentrations. **(A–C)** show the three LAMP amplification curves, respectively; **(D)**. Stability of the three LAMP experiments.

Stability is also a crucial performance indicator of detection methods and systems. The system uses high, medium, and low concentration standard samples that are repeated three times to ensure reproducibility. The amplification curves of the three assays are shown in Figures 5A,C. From the overall perspective of the curved shape, the high, medium, and low concentrations of the three experiments could be amplified normally, the curve shape similarity was high, and the reproducibility between batches was high concentration > medium concentration > low concentration. We calculated the SD and CV values of the Tt values for the three experiments at three concentrations. As shown in Figure 5D, the CVs of the high, medium, and low concentrations were 8.69, 3.02, and 9.05%, respectively. The performance was medium concentration > high concentration > low concentration. The above CV values were <10%, indicating that the overall amplification efficiency of the system was reproducible. Additionally, the detection results of the repeatable experiments on commercial PCR instruments were consistent with those in this system, so the stability of this system for point-of-care diagnosis of norovirus meets the requirements and could be applied to real-time qualitative monitoring of the virus, with high specificity, sensitivity, and rapidity.

### 4.4 Rt-LAMP and RT-qPCR for the detection of clinical samples

**FIGURE 6:**
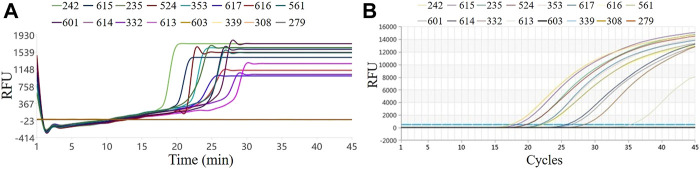
Results of clinical samples by RT-LAMP and RT-qPCR. **(A)** RT-LAMP on our system; **(B)** RT-qPCR on the commercial instrument.

The results of temperature, uniformity, sensitivity, and reproducibility show that the system can be used for pathogen detection in a stable, fast, convenient, and portable manner. We further verified the performance of the system to test actual samples. We tested 45 clinical norovirus diarrhea samples, including 12 positive and 33 negative samples. We selected three negative and 12 positive samples for testing in this system, and the results are shown in Figure 6. Figure 6A represents the RT-LAMP amplification curve, and Figure 6B is the RT-qPCR amplification curve. The Ct value of RT-qPCR curve and the Tt value of the RT-LAMP curve were in the same order, indicating that the RT-LAMP and RT-qPCR detection results of this batch of clinical samples on the system are consistent. From the comparison between the Tt value and the time to reach the plateau phase, it was revealed that LAMP could reach the plateau phase within 5 min after the Tt value starts, while qPCR could reach the plateau phase after at least 15 cycles. Therefore, LAMP has an absolute advantage in the overall response time, making this system ideal for rapid on-site pathogen detection.

We discovered that the uniformity and stability of this system were not significantly different from those of commercial equipment after summarizing the above performance test and evaluation results. The linear range detected by this system was 10^0^–10^6^ copies/μL, and the sensitivity of the system can be similar to that of commercial PCR instruments. Furthermore, the detection of 45 clinical samples was consistent with the qPCR and clinical results, and the accuracy could reach 100%. Another outstanding performance of this system is that it could significantly reduce the detection time and improve the amplification efficiency when detecting pathogens. For raw data on performance testing, please check Supplementary Tables S1, S2 and Supplementary Figures S1–S4 in the Supplementary Materials.

## 5 Conclusion

Summarily, in this study, we successfully developed a real-time fluorescent RT-LAMP detection system and applied it to pathogen detection. The whole system is compact and portable, and it can rapidly and effectively detect pathogens. For the norovirus detection, the entire process could be completed within 45 min, and the system can achieve the theoretical sensitivity of LAMP detection, test results could be determined within 35 min, and quantitative testing was possible. The overall uniformity and reproducibility of the system were good, and the detection results of clinical samples were consistent with the RT-qPCR and clinical results. Moreover, the cost is much lower than that of commercial qPCR, and it uses conventional reagent consumables and has 16 sample detection holes, making it very suitable for areas where medical conditions are relatively scarce or need on-site environments. It can also be used to detect various epidemiological and unexpected diseases such as novel coronavirus, monkeypox virus, African swine fever, etc. Thus, these show that this system has the characteristics of portability, speed, and accuracy of a POCT.

## Data Availability

The original contributions presented in the study are included in the article/Supplementary Material, further inquiries can be directed to the corresponding author.
