# Supplementary figures and images for: Novel miniaturized fluorescence loop-mediated isothermal amplification detection system for rapid on-site virus detection
Source: Front Bioeng Biotechnol. 2022 Aug 24;10:964244. doi: 10.3389/fbioe.2022.964244 (PMC9448916; doi:10.3389/fbioe.2022.964244)

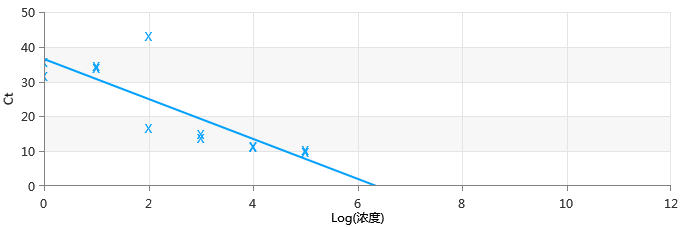

Supplement: Supplementary file 2 [file Image4.PNG]

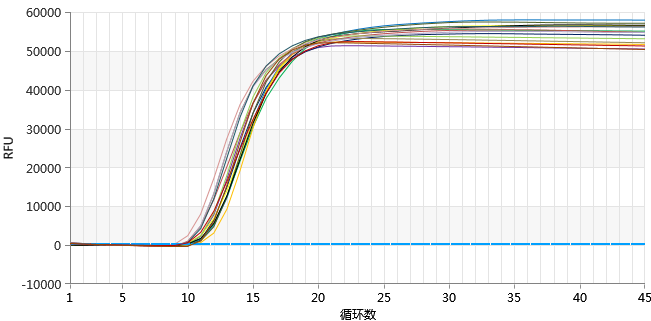

Supplement: Supplementary file 3 [file Image2.PNG]

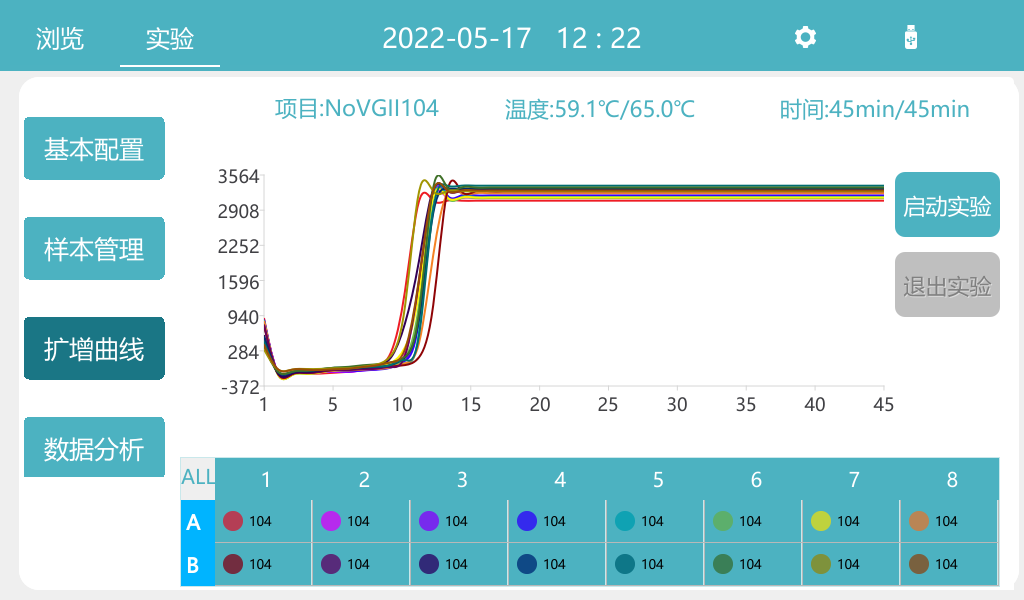

Supplement: Supplementary file 5 [file Image1.PNG]

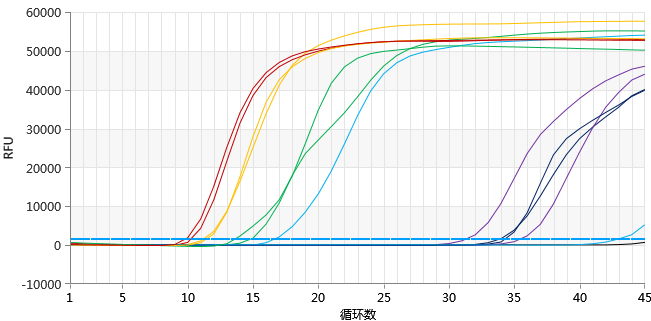

Supplement: Supplementary file 6 [file Image3.PNG]
